# Au@CuS Nanoshells for Surface-Enhanced Raman Scattering Image-Guided Tumor Photothermal Therapy with Accelerated Hepatobiliary Excretion

**DOI:** 10.3390/pharmaceutics16081089

**Published:** 2024-08-20

**Authors:** Sihang Zhang, Sheng Yu, Jingwen Sun, Teng Huang, Hongzheng Lin, Zhe Li, Zeyu Xiao, Wei Lu

**Affiliations:** 1 School of Pharmacy & Minhang Hospital, Key Laboratory of Smart Drug Delivery, Ministry of Education & State Key Laboratory of Molecular Engineering of Polymers, Fudan University, 826 Zhangheng Road, Shanghai 201203, China; 2 Department of Pharmacology and Chemical Biology, Key Laboratory of Cell Differentiation and Apoptosis of Chinese Ministry of Education, Shanghai Jiao Tong University School of Medicine, 280 South Chongqing Road, Shanghai 200025, China; 3 Quzhou Fudan Institute, 108 Minjiang Avenue, Quzhou 324002, China

**Keywords:** gold nanoparticles, copper sulfide (CuS), hepatobiliary excretion, surface-enhanced Raman scattering (SERS), photothermal therapy (PTT)

## Abstract

Gold-based nanoparticles for surface-enhanced Raman scattering (SERS) imaging show great potential for precise tumor detection and photothermal therapy (PTT). However, the metabolizability of gold nanoparticles (Au NPs) raises big concerns. Herein, we designed a core-shelled nanostructure of copper sulfide (CuS)-coated Au NPs with surface pegylation (PEG-Au@CuS NSs). The excreted Au in the gallbladders at 1 h and 4 h in mice injected with PEG-Au@CuS NSs was 8.2- and 19.1-fold of that with the pegylated Au NPs (PEG-AuNPs) of the same Au particle size, respectively. By loading the Raman reporter 3,3′-Diethylthiatricarbocyanine iodide (DTTC) in the core–shell junction of PEG-Au@CuS NSs, the PEG-Au-DTTC@CuS NSs exhibited the Raman signal-to-noise (S/N) ratio of 4.01 after 24 h of intravenous (IV) injection in the mice bearing an orthotopic CT26-Luc colon tumor. By contrast, the DTTC-coated PEG-AuNPs (PEG-Au-DTTC NPs) achieved an S/N ratio of 2.71. Moreover, PEG-Au-DTTC@CuS NSs exhibited an increased photothermal conversion effect compared with PEG-Au-DTTC NPs excited with an 808-nm laser. PEG-Au-DTTC@CuS NSs enabled intraoperative SERS image-guided photothermal therapy for a complete cure of the colon tumor-bearing mice. Our data demonstrated that the PEG-Au-DTTC@CuS NSs are promising intraoperative Raman image-guided theranostic nanoplatform with enhanced hepatobiliary excretion.

## 1. Introduction

SERS imaging has attracted great attention for the potential applications of diagnosis and therapeutic guidance owing to the ultrasensitive Raman signals [1,2,3,4]. Au NPs are the most commonly used substrate materials for SERS, which can significantly enhance Raman signals by the local surface plasma resonance (LSPR) effect [5]. Besides, the LSPR effect enables the photothermal conversion effect of Au NPs [6,7,8,9,10]. Therefore, the Au NP-based SERS exhibit a promising strategy to accurately locate tumors for the precise PTT [11,12,13]. Oseledchyk et al. reported a robust ratiometric Raman imaging approach using targeted and nontargeted gold-based SERS NP multiplexing, which can detect tumor lesions as small as 370 μm [14]. Chen et al. proposed a gold-based SERS imaging probe with a superior photothermal ablation effect on U87 glioma cells, and performed a SERS image-based real-time sensitive monitoring of microscopic temperature during PTT unaffected by the cellular microenvironment [15].

Despite their wide application in clinical diagnostics, therapeutics, or their combination for theranostics, the Au NP-based SERS are still in the early stage of clinical development. No product or technology has been marketed so far [16,17], which is mainly limited by the in vivo clearance of Au NPs and the stability of Raman tags [18]. Au NPs are considered to be rarely biodegradable and usually accumulate for a long time in vivo, which raises further safety issues and limits their clinical translation [19,20,21,22]. Our previous study demonstrated that only 3.98% of the intravenously injected 40 nm-sized Au NPs could be excreted within 30 days post-injection [23]. Besides, in vivo stability is a serious concern for the Raman reporter-modified Au NPs. After the intravenous injection, competitive adsorption of the various components to Raman reporter molecules would take place in the biological environment, which may lead to the quenching of the SERS signal [24]. Thus, well-designed protective layers such as bovine serum albumin (BSA), polyethylene glycol (PEG), liposomes, and silica are necessary for the biostability of SERS probes [25,26].

CuS is a p-type semiconductor material with excellent photoelectric properties and good biocompatibility, which has been extensively applied in the fields of tumor treatment, drug delivery, in vivo imaging, etc. [27,28,29]. We have previously illustrated that the injected pegylated CuS nanoparticles (CuS NPs) are eliminated through both hepatobiliary (67 percent of injected dose, % ID) and renal (23% ID) excretion within 30 days post-injection [30]. In addition, CuS is able to facilitate the hepatocyte exocytosis and biliary excretion of Au NPs by copper-transporting ATPase ATP7B, through the design of different CuS-Au nanoconjugates [30]. Besides, CuS NPs can absorb laser energy in near-infrared (NIR) and mid-infrared light and convert it into heat for in vivo photoacoustic imaging and tumor photothermal ablation [31,32,33].

In this work, we prepared a core-shelled structure of Au NPs coated with a CuS layer followed by surface pegylation (PEG-Au@CuS NSs). Compared with Au NPs of the same Au particle size, the PEG-Au@CuS NSs showed significantly accelerated hepatobiliary excretion and stronger absorption in the NIR window ranging from 750 nm to 1100 nm, exhibiting a promising photothermal conversion effect in vitro and in vivo. By loading the Raman reporter molecule DTTC, the PEG-Au-DTTC@CuS NSs showed remarkable SERS signals at 506 cm^−1^ and 847 cm^−1^. In mice bearing the orthotopic CT26-Luc colon tumor model, this nanoplatform outperformed the DTTC-loaded Au NPs (PEG-Au-DTTC NPs) in terms of in vivo Raman imaging sensitivity. These advantages enabled the intraoperative Raman imaging to precisely distinguish colon tumors from normal tissue, guiding the photothermal therapy with a complete cure of the tumor-bearing mice.

## 2. Materials and Methods

### 2.1. Materials

Chloroauric acid trihydrate (HAuCl_4_·4H_2_O), dimethyl sulfoxide (DMSO), sodium citrate, acetic acid copper salt hydrate (Cu(Ac)_2_·2H_2_O), sodium sulfide nonahydrate (Na_2_S·9H_2_O), 3-(4,5-Dimethylthiazol-2-yl)-2,5-diphenyltetrazolium bromide (MTT), and sodium hydroxide (NaOH) were purchased from Sinopharm Chemical Reagent Co., Ltd. (Shanghai, China). Thiolated polyethylene glycol_5000_-methoxy (SH-PEG_5000_-methoxy) was purchased from Seebio Biotech Co., Ltd. (Shanghai, China). 3,3′-Diethylthiatricarbocyanine iodide (DTTC) was purchased from Macklin Biochemical Technology Co., Ltd. (Shanghai, China). Polyvinylpyrrolidone (PVP) and ascorbic acid were purchased from Sigma-Aldrich (Saint Louis, MO, USA). William’s E medium was purchased from Basalmedia Technologies Co., Ltd. (Shanghai, China). All reagents were of analytical purity and used without further purification.

### 2.2. Cells and Animals

Mouse colon cancer CT26 cell line stably expressing luciferase CT26-Luc (CT26.WT-Fluc-Neo) was obtained from Imanis Life Science (Rochester, MN, USA). Cells were cultured in Roswell Park Memorial Institute (RPMI)-1640 with 10% (*v*/*v*) fetal bovine serum (FBS, Meilunbio, Dalian, China), penicillin (100 μg/mL), and streptomycin (100 μg/mL) at 5% CO_2_ and 37 °C.

BALB/c mice (male, 6–8 weeks) were ordered from Shanghai Slac Biological Co., Ltd. (Shanghai, China) and housed under specific pathogen-free (SPF) conditions with free access to water and food. All the animals were allowed to adapt to the environment before experiments. All the animal experiments were performed under the guidance of Institutional Animal Care and Use Committee (IACUC) of School of Pharmacy, Fudan University.

### 2.3. Preparation of Nanoparticles

#### 2.3.1. Preparation of PEG-AuNPs or PEG-Au-DTTC NPs

The Au NPs were prepared according to the literature report [34]. For the modification of PEG, the synthesized Au NPs were centrifuged at 15,000 rpm for 15 min at room temperature, and then resuspended in water containing SH-PEG_5000_-methoxy (1 mg/mL) overnight at room temperature to obtain PEG-AuNPs. For the preparation of PEG-Au-DTTC NPs, 700 μL of the Raman reporter DTTC (10 μg/mL in DMSO) and 500 μL of SH-PEG_5000_-methoxy (1 mg/mL) were added to 20 mL of the as-synthesized Au NPs in sequence under vigorous stirring. After 5 min, the solution of PEG-AuNPs labeled with DTTC was centrifuged at 11,000 rpm for 15 min to obtain PEG-Au-DTTC NPs.

#### 2.3.2. Preparation of PEG-Au@CuS NSs or PEG-Au-DTTC@CuS NSs

PEG-Au@CuS NSs were prepared according to the previous procedures [35]. Typically, 10 mL PVP solution (50 μg/mL) was added to 100 mL of synthesized Au NP solution (1.5 mg/mL of Au) under stirring for 10 min. Cu(Ac)_2_ (0.1 M, 250 μL) was added under stirring for 5 min, followed by addition of NaOH (1 M, 700 μL). After 2 min, ascorbic acid (0.1 M, 1.5 mL) was added. The mixture was left undisturbed for 30 min at room temperature. Then, the temperature was increased to 90 °C. Under stirring, Na_2_S (0.1 M, 250 μL) was added. After 2 h, the system was naturally cooled down to room temperature. The product was purified by centrifugation (11,000 rpm, 15 min) and washed with deionized water twice. Au@CuS NSs were then resuspended in water containing SH-PEG_5000_-methoxy (1 mg/mL) overnight at room temperature to obtain PEG-Au@CuS NSs.

For the preparation of PEG-Au-DTTC@CuS NSs, the as-synthesized PEG-Au-DTTC NPs were used as the core instead of the Au NPs. The PEG-Au-DTTC NPs were coated with CuS shell followed by pegylation with the same method as PEG-Au@CuS NSs.

### 2.4. Characterization of Different Nanoparticles

The morphology of NPs was observed by transmission electron microscopy (TEM) (FEI Tecnai G2 F20 S-Twin, Hillsboro, OR, USA). Zeta potential and size distribution of the synthetic nanoparticles were measured by a dynamic light scattering (DLS) instrument (Malvern Nanozetasizer, Worcester, UK). Ultraviolet-visible-near-infrared (UV-vis-NIR) absorption spectra were detected on a Lambda 365 spectrophotometer (PerkinElmer, Waltham, MA, USA).

### 2.5. Raman Stability in FBS

To determine the serum stability of Raman signals, PEG-Au-DTTC NPs and PEG-Au-DTTC@CuS NSs (both 10 μg/mL of Au) were incubated in FBS at 37 °C and Raman signals were determined at 0 and 24 h, respectively.

### 2.6. In Vitro Cytotoxicity and Photothermal Ablation Effect of PEG-Au-DTTC@CuS NSs

NIH 3T3 cells obtained from the American Type Culture Collection (ATCC, Manassas, VA, USA) were used for the in vitro cytotoxicity evaluation of PEG-Au-DTTC@CuS NSs via MTT assay. The cells (8 × 10^3^ per well) were cultured in 96-well microplates for 24 h before the experiment. The cells were incubated with a series of concentrations of PEG-Au-DTTC@CuS NSs for 24 h. Replaced with fresh culture medium, 20 µL of MTT solution (5 mg/mL) was added. After 2 h of incubation, the supernatant was replaced with 150 µL of DMSO. The absorbance was measured at 570 nm and 690 nm via Bio-Rad 550 microplate reader (Bio-Rad, Hercules, CA, USA).

For evaluation of photothermal ablation effect, CT26-Luc cells (8 × 10^3^ per well) were cultured in 96-well microplates for 24 h before experiment. The cells were incubated with a series of concentrations of PEG-Au-DTTC@CuS NSs in serum-free medium for 4 h. Replaced with fresh culture medium, the cells received an 808-nm laser irradiation for 5 min (1.0 W/cm^2^). The cells were continuously cultured for 12 h. Cell viability was determined with MTT as mentioned above.

### 2.7. Isolation of Primary Mouse Hepatocytes and Kupffer Cells

Primary mouse hepatocytes and Kupffer cells were isolated by a two-step collagenase perfusion according to the literature report [30,36].

### 2.8. Quantification of Au in Mouse Hepatocytes and Kupffer Cells Following Incubation with Different Nanoparticles

The 12-well plate (Corning Inc., Corning, NY, USA) was coated with 50 μg/mL of type I collagen (Yeasen, Shanghai, China) in advance. Mouse primary hepatocytes were seeded according to our precious protocol 2 days before the experiment [30]. For the analysis of cellular Au content, the medium was replaced with 500 μL of medium containing PEG-Au@CuS NSs or PEG-AuNPs (50 μg/mL of Au) for 5 min at 37 °C. After being washed, fresh medium was added for exocytosis. At 5, 15, 35, or 65 min, the medium containing nanoparticles was removed and cells were quickly washed with William’s E medium. Then, 100 μL of RIPA lysis buffer (Meilunbio, Dalian, China) was added and cell lysates were collected. After centrifugation at 15,000 rpm for 10 min, the pellet was digested with aqua regia and quantified by inductively coupled plasma optical emission spectrometer (ICP-OES, Thermo Fisher Scientific, Waltham, MA, USA) analysis. The protein content of the cell lysates was analyzed by BCA protein assay kit (Yeasen, Shanghai, China).

Mouse Kupffer cells were seeded in 48-well plate 24 h before the experiment. The study of uptake and exocytosis of nanoparticles in Kupffer cells was conducted using the above-described procedure for those in the hepatocytes.

% dose/mg protein represents the cellular Au content, which is calculated as follows:

(1) % dose/mg protein=cellular Au per wellAu added μg per wellcellular protein mg per well×100%

% Au exocytosis_0−t_ represents the decreased percentage of cellular Au content during the exocytosis period from 0 to t min and is calculated as follows:

(2) $${\%\;Au\;exocytosis}_{0-t}=\left(1-\frac{\%\;dose/mg\;{protein}_{t+5}}{\%\;dose/mg\;{protein}_{5}}\right)\times 100\%$$

### 2.9. Excretion of Nanoparticles into Gallbladders of Mice

BALB/c mice were divided into 3 groups (*n* = 4). One group of mice injected with PBS was used as the blank control. The other two groups were IV-injected with PEG-Au@CuS NSs or PEG-AuNPs, respectively (40 mg/kg of Au). The gallbladder of each mouse was collected at 1 h or 4 h post-injection. Each gallbladder was weighed and digested with aqua regia and then the Au content was quantified by ICP-OES. In a parallel study, the bile collected from gallbladder was centrifugated at 15,000 rpm for 10 min. Then, the pellet was resuspended in water and directly deposited on the nickel grid and analyzed with scanning transmission electron microscopy (STEM, JEOL, JEM 2100, Tokyo, Japan) and energy-dispersive X-ray spectroscopy (EDX, Oxford Instruments, INCA xsight, Oxfordshire, UK).

### 2.10. In Vitro Photothermal Conversion Effect

The aqueous solutions of PEG-Au@CuS NSs and PEG-AuNPs were irradiated under an 808 nm laser (1.0 W/cm^2^) for 10 min, respectively. The temperature of the solution was recorded every second by a digital thermometer with a thermocouple probe.

To investigate the photothermal conversion efficiency, PEG-Au@CuS NSs or PEG-AuNPs (50 μg/mL of Au) were irradiated for 10 min (808 nm, 1.0 W/cm^2^) to reach a steady state temperature, then the laser was turned off and the solution naturally cooled down. The photothermal efficiency was then calculated according to the Equation (S1) (Supplementary Method).

### 2.11. Establishment of Tumor Models

For the establishment of orthotopic CT26-Luc colon tumor model, CT26-Luc cells (2 × 10^6^) in 15 μL of PBS were injected into the subserosal layer of mouse cecum according to our previously reported procedures [37].

### 2.12. Raman Measurements

In vitro and in vivo Raman spectra were acquired by inVia Raman microscope (Renishaw, Wotton-under-Edge, Gloucestershire, UK). The Raman images were analyzed by a signal-to-baseline algorithm (WiRE 4.3 software, Renishaw, UK). To detect the Raman signal of PEG-Au-DTTC@CuS NSs and PEG-Au-DTTC NPs in an aqueous solution, the Raman spectra were acquired with a 785 nm laser at 84.5 mW with a 1 s acquisition time and a 5× objective.

For Raman imaging of colon tumor, at 4-day post-tumor establishment, the BALB/c mice were IV-injected with PEG-Au-DTTC NPs or PEG-Au-DTTC@CuS NSs in PBS solution (both 40 mg/kg of Au). At 24 h post-injection, the mice received an abdominal skin incision under anesthesia to expose the cecum for Raman imaging. In vivo Raman imaging was obtained with a 785 nm laser excitation with a power of 84.5 mW, 5× objective, an acquisition time of 2 s, and one-time accumulation using StreamLine high-speed acquisition mode. Characteristic peaks at 506 and 847 cm^−1^ for PEG-Au-DTTC NPs or PEG-Au-DTTC@CuS NSs were selected for the image processing, respectively.

### 2.13. Raman Image-Guided Tumor Photothermal Therapy

BALB/c mice bearing orthotopic CT26-Luc tumors were randomly divided into 3 groups (*n* = 5): (1) mice receiving PEG-Au-DTTC@CuS NSs and Raman image-guided PTT, (2) mice receiving PEG-Au-DTTC@CuS NSs and PTT based on visual inspection (conventional PTT), and (3) mice injected with PBS as control. For groups (1) and (2), mice were IV-injected with PEG-Au-DTTC@CuS NSs (40 mg/kg of Au) 24 h before PTT treatment under 808-nm irradiation (1.0 W/cm^2^) for 10 min. Otherwise, for mice receiving Raman image-guided PTT, tumors were collected at 24 h after PTT and sectioned for hematoxylin and eosin (H&E) staining.

The growth of colon tumor was monitored by IVIS Spectrum Imaging System (Caliper Life Sciences, Hopkinton, MA, USA) after intraperitoneal injection of D-luciferin (15 mg/mL, 200 µL). The tumor and normal colon tissue were collected at the end point of the experiment, and sectioned for H&E staining.

### 2.14. Statistical Analysis

Statistical analysis was performed using Graphpad Prism 8.0. All the results were presented as mean ± SD. The data were analyzed by two-way ANOVA with Sidak’s post-hoc test for multiple groups.

## 3. Results

### 3.1. Characterization of PEG-AuNPs and PEG-Au@CuS NSs

**Figure 1:**
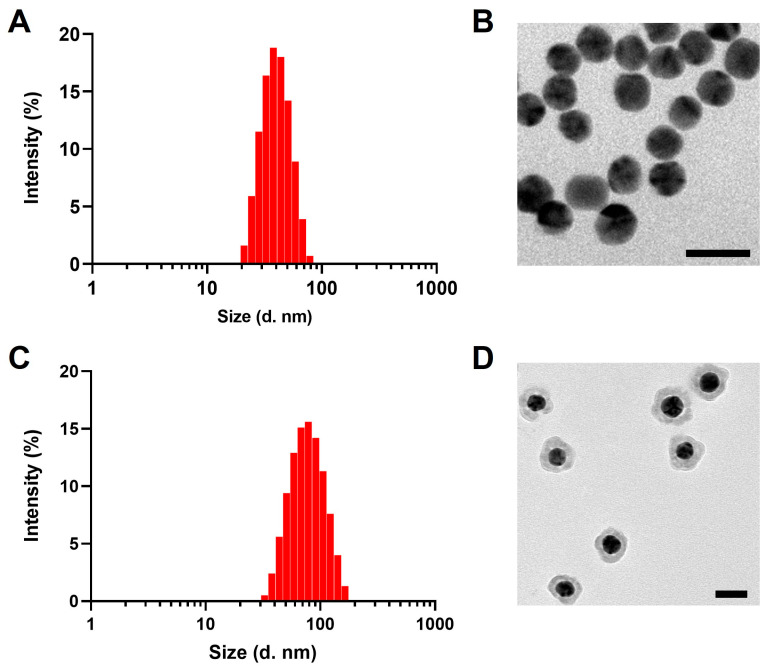
Size distribution and TEM images of PEG-AuNPs (**A**,**B**) and PEG-Au@CuS NSs (**C**,**D**), respectively. Bar: 50 nm.

Figure 1 shows the TEM images and distribution of the hydrodynamic diameters of the PEG-AuNPs and PEG-Au@CuS NSs, respectively. The as-prepared PEG-AuNPs were well-dispersed and showed a relatively uniform spherical shape with an average hydrodynamic diameter of 35 nm with a polymer dispersity index (PDI) of 0.16 (Figure 1A). After the CuS shell growth, the diameter of PEG-Au@CuS NSs increased to 70 nm with a PDI of 0.13 (Figure 1C) and maintained a stable size distribution after incubation in 10% FBS for 24 h (Figure S1).

### 3.2. Hepatobiliary Excretion of PEG-AuNPs and PEG-Au@CuS NSs

**Figure 2:**
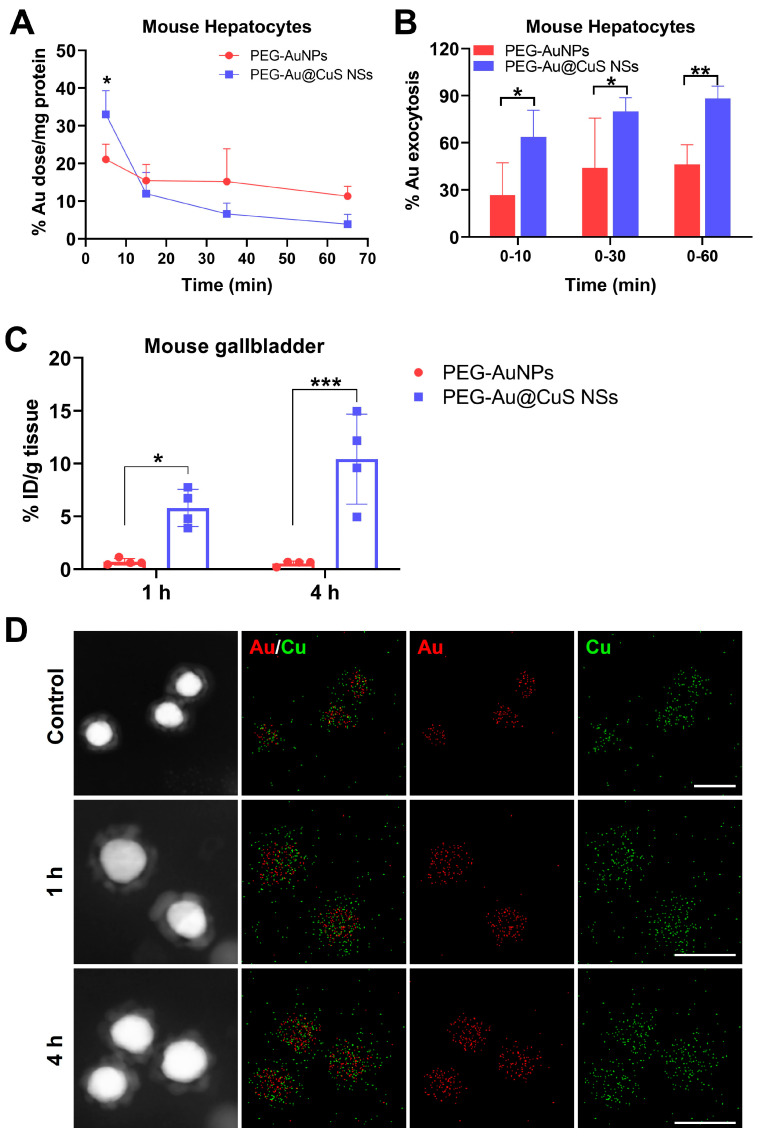
Cellular Au (**A**) and percent Au exocytosis (**B**) from the primarily cultured mouse hepatocytes following the 5 min uptake of PEG-AuNPs or PEG-Au@CuS NSs, respectively. (**C**) Quantitative analysis of Au excreted into the gallbladder of mice at 1 h or 4 h following IV injection of PEG-AuNPs or PEG-Au@CuS NSs (both 40 mg/kg of Au). (**D**) STEM/EDX analysis of PEG-Au@CuS NSs in bile samples of BALB/c mice at 1 h and 4 h following IV injection. Control, PEG-Au@CuS NSs in water. Bar: 50 nm. Data are presented as mean ± SD (*n* = 4). Statistical significance was calculated by two-way ANOVA with Sidak’s post-hoc test. * *p* < 0.05, ** *p* < 0.01, *** *p* < 0.001.

We quantitatively analyzed the cellular content and the exocytosis percentage of PEG-AuNPs and PEG-Au@CuS NSs in the cultured primary mouse hepatocytes. The cells were incubated with the nanoparticles for a 5 min uptake, followed by the replacement with fresh medium for exocytosis. As shown in Figure 2A, after the 5 min uptake, the cellular content of Au in the PEG-Au@CuS NSs-treated hepatocytes was 1.6-fold that of the PEG-AuNPs. Whereas, within 30 min or 60 min of exocytosis, a lower amount of Au remained in PEG-Au@CuS NSs-treated hepatocytes. The percentage of Au exocytosis of PEG-Au@CuS NSs and PEG-AuNPs from the hepatocytes in 60 min was 88% and 46%, respectively (Figure 2B). Thus, the PEG-Au@CuS NSs revealed facilitated exocytosis in hepatocytes in comparison with the PEG-AuNPs.

Besides, we evaluated the uptake and exocytosis of PEG-Au@CuS NSs by the primary mouse Kupffer cells in comparison with that of PEG-AuNPs. The results showed a significantly higher uptake of Au in the PEG-Au@CuS NSs-treated Kupffer cells than that of the PEG-AuNPs-treated cells at 5 min following the incubation (Figure S2A). However, the Au exocytosis rate in the PEG-Au@CuS NSs-treated Kupffer cells was similar to that of the PEG-AuNPs-treated cells within 60 min of the exocytosis period (Figure S2B). After the initial 5 min, about 53.8% of PEG-Au@CuS NSs were phagocytized by Kupffer cells, compared to 33.0% by hepatocytes (Figure 2A and Figure S2A). However, within the 60 min study, the Au exocytosis rate of PEG-Au@CuS NSs was slower in Kupffer cells than that of hepatocytes (71.1 vs 88.3%, Figure 2B and Figure S2B). This resulted in 15.6% remaining in Kupffer cells while only 3.9% remained in hepatocytes (Figure 2A and Figure S2A). Collectively, these results demonstrated that Kupffer cells exhibited a higher uptake capacity of PEG-Au@CuS NSs than hepatocytes, while maintaining a relatively lower excretion rate.

According to the biodistribution study of PEG-Au@CuS NSs and PEG-AuNPs at 4 h following the IV injection, the concentration of PEG-Au@CuS NSs was much lower in the blood at 4 h post-injection, indicating a much faster blood clearance (Figure S3). Considerably, the Au content in gallbladders from the mice treated with PEG-Au@CuS NSs was 8.2- and 19.1-fold higher than that of the PEG-AuNPs-treated mice at 1 h and 4 h post-injection, respectively (Figure 2C). EDX analysis confirmed the distribution of Au and Cu elements from the PEG-Au@CuS NSs in bile at 1 h and 4 h post-injection, respectively (Figure 2D). These results demonstrated that PEG-Au@CuS NSs increased hepatocyte exocytosis and biliary excretion following the CuS coating on Au NPs, showing potential for in vivo application.

### 3.3. UV Absorption and In Vitro Photothermal Effect

**Figure 3:**
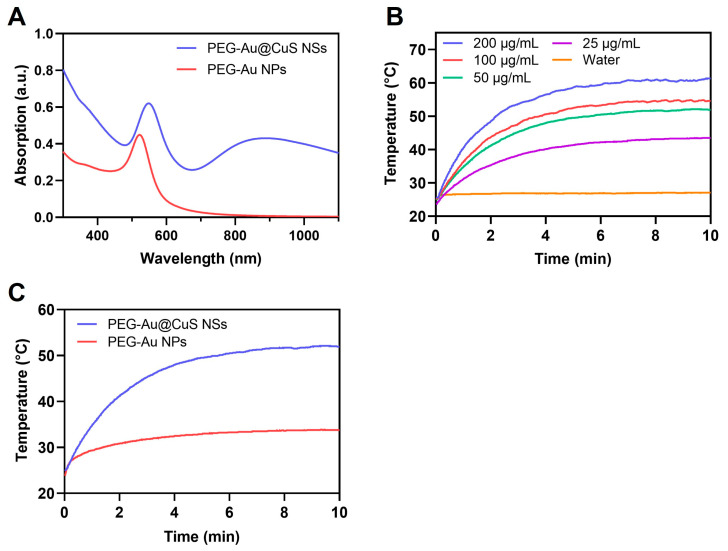
(**A**) UV absorption spectra of PEG-Au@CuS NSs and PEG-AuNPs. (**B**) Photothermal effect of PEG-Au@CuS NSs at different concentrations or water under 808 nm laser irradiation (1.0 W/cm^2^) for 10 min. (**C**) Photothermal effect of PEG-Au@CuS NSs and PEG-AuNPs (both 50 μg/mL of Au) under 808 nm laser irradiation (1.0 W/cm^2^) for 10 min, respectively.

The absorption spectra of PEG-AuNPs and PEG-Au@CuS NSs are shown in Figure 3A. The peak of PEG-AuNPs at 521 nm was red-shifted to 548 nm with the enhanced peak intensity following coating with the CuS layer (PEG-Au@CuS NSs). The PEG-Au@CuS NSs exhibited a broad absorption in the NIR region and peaked at 880 nm, favoring their efficient photothermal conversion following the 808-nm laser irradiation for deep tissue penetration. The increases in the temperature of PEG-Au@CuS NSs and PEG-AuNPs solution were both dependent on the concentration of the nanoparticles under the irradiation of the 808 nm laser at a power of 1.0 W/cm^2^ (Figure 3B and Figure S4). Compared with PEG-AuNPs, the PEG-Au@CuS NSs solution at the same concertation of Au generated a higher temperature upon NIR laser irradiation at the same power density. Specifically, following irradiation with the 808 nm laser (1.0 W/cm^2^) for 10 min, the temperature of the Au@CuS NSs solution (50 μg/mL of Au) reached 52 °C, while the temperature of the Au NPs (50 μg/mL of Au) reached 34 °C under identical conditions (Figure 3C). The photothermal conversion efficiency of PEG-Au@CuS NSs and PEG-AuNPs was calculated to be 20.0% and 35.2%, respectively (Figure S5). The lower photothermal conversion efficiency of PEG-Au@CuS NSs than PEG-AuNPs was because the absorption of PEG-AuNPs was much lower than that of PEG-Au@CuS NSs at 808 nm (0.0425 vs 0.4). Despite the higher photothermal conversion efficiency of PEG-AuNPs, PEG-Au@CuS NSs raised much higher temperatures of the solution than PEG-AuNPs at the same concentration of Au (Figure S5A). Collectively, PEG-Au@CuS NSs showed greater potential for tumor PTT.

### 3.4. Raman Spectra of the DTTC-Loaded Nanoparticles

**Figure 4:**
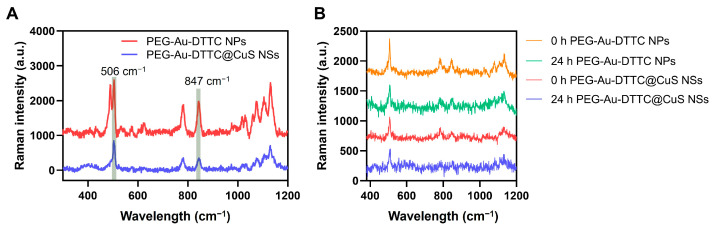
(**A**) Raman spectra of PEG-Au-DTTC NPs and PEG-Au-DTTC@CuS NSs (both 20 μg/mL of Au). (**B**) Raman spectra of PEG-Au-DTTC NPs and PEG-Au-DTTC@CuS NSs at 506 cm^−1^ before (0 h) and after the incubation with FBS at 37 °C for 24 h (both 10 μg/mL of Au).

By loading the Raman reporter DTTC, the synthesized PEG-Au-DTTC NPs and PEG-Au-DTTC@CuS NSs exhibited a zeta potential of −3.47 ± 1.44 mV and −11.4 ± 0.35 mV, respectively (Figure S6). Both nanoparticles displayed fingerprint peaks at 506 cm^−1^ and 847 cm^−1^ (Figure 4A), which were chosen for further Raman imaging processing. For the PEG-Au-DTTC NPs, the DTTC molecules were modified via the Au-S bond on the surface of Au NPs. For the PEG-Au-DTTC@CuS NSs, DTTC was further embedded in the core-shelled junction, where the CuS layer can prevent detachment or leakage of the Raman reporter in the in vivo environment. Thus, the Raman intensity of PEG-Au-DTTC NPs NSs at 506 cm^−1^ was decreased by 28.3% after a 24 h incubation in FBS compared with that of 0 h (Figure 4B). However, the Raman intensity of PEG-Au-DTTC@CuS NSs at 506 cm^−1^ remained almost unchanged. Moreover, the nanoparticles exhibited negligible cytotoxicity after incubation with NIH 3T3 cells for 24 h (Figure S7), indicating the biocompatibility for the in vivo application.

### 3.5. In Vivo Photothermal Effect

**Figure 5:**
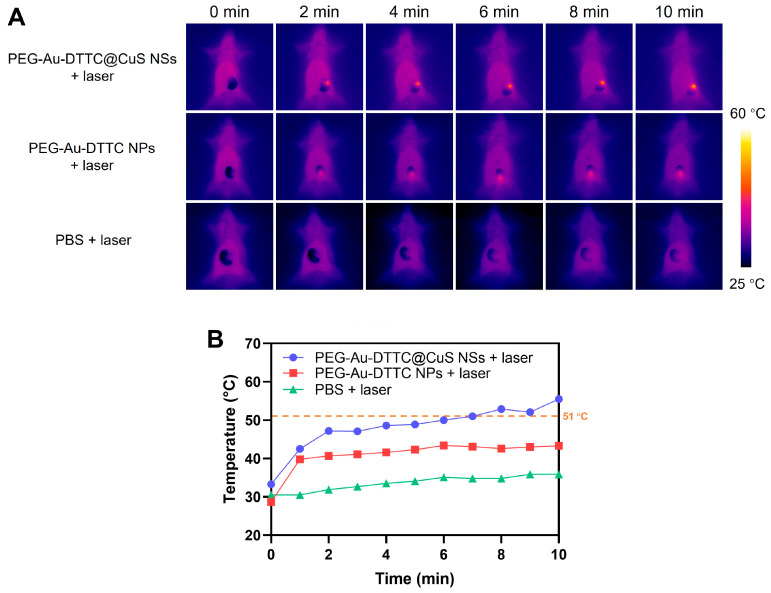
(**A**) Infrared thermal images of the tumor-bearing mice receiving different treatment under 808 nm laser irradiation (1.0 W/cm^2^, 10 min) for different times. The mice were IV-injected with PBS, PEG-Au-DTTC@CuS NSs, or PEG-Au-DTTC NPs (both 40 mg/kg of Au) 24 h before the laser treatment. (**B**) The plots of temperature in the tumor over time from the images of (**A**).

At 24 h post-injection of PEG-Au-DTTC@CuS NSs, PEG-Au-DTTC NPs, or PBS, the infrared thermal images and corresponding temperature curves of the tumor-bearing mice under anesthesia with the tumor sites exposed were recorded following the laser irradiation over time (Figure 5). For the tumors of mice injected with PEG-Au-DTTC@CuS NSs, the temperature of the tumor area was raised to 51 °C after NIR laser irradiation for 7 min (808 nm, 1.0 W/cm^2^). The threshold of tissue thermal ablation was reported to be 51 °C for 100 s [37,38,39]. However, there was a less increase of temperature in the PEG-Au-DTTC NPs group under the same irradiation conditions with a terminal temperature of 43 °C. The minimal time duration to induce a tissue thermal ablation at 43 °C was calculated to be 120 min [40]. The results of the comparative study demonstrated that the PEG-Au-DTTC@CuS NSs exhibited much higher in vivo photothermal conversion efficiency than PEG-Au-DTTC NPs. Moreover, PEG-Au-DTTC@CuS NSs exerted a photothermal ablation effect on tumor cells following the NIR laser irradiation in a concentration-dependent manner (Figure S8).

### 3.6. In Vivo Raman Imaging

**Figure 6:**
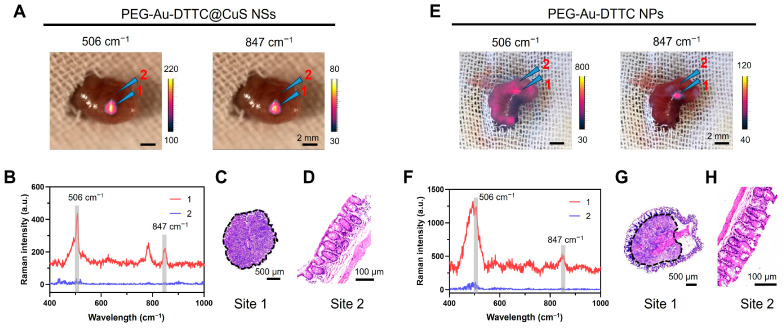
(**A**) Raman image of tumor of mice bearing orthotopic CT26-Luc tumor at 24 h post-injection of PEG-Au-DTTC@CuS NSs (40 mg/kg of Au). Arrow 1: tumor, arrow 2: normal tissue. (**B**) Raman spectra of different sites in (**A**). (**C**) H&E staining confirmed the tissue sections from site 1 in (**A**). (**D**) H&E staining confirmed the tissue sections from site 2 in (**A**). (**E**) Raman image of tumor of mice bearing orthotopic CT26-Luc tumor at 24 h post-injection of PEG-Au-DTTC NPs (40 mg/kg of Au). Arrow 1: tumor, arrow 2: normal tissue. (**F**) Raman spectra of different sites in (**E**). (**G**) H&E staining confirmed the tissue sections from site 1 in (**E**). (**H**) H&E staining confirmed the tissue sections from site 2 in (**E**).

The tumor-bearing mice were IV-injected with PEG-Au-DTTC NPs or PEG-Au-DTTC@CuS NSs (both 40 mg/kg of Au). After 24 h, the intraoperative Raman imaging was performed by surgical exposure of the cecum with the tumor. According to the characteristic Raman signals of PEG-Au-DTTC@CuS NSs at 506 cm^−1^ or 847 cm^−1^, Raman imaging could effectively distinguish the tumor area from the normal cecum tissue (Figure 6A,B) with the Raman S/N ratio of 4.01 at 847 cm^−1^. This was confirmed by H&E staining of the resected tissues that the Raman signal-positive area (arrow 1) and Raman signal-negative area (arrow 2) were tumor and colon, respectively (Figure 6C,D). Comparatively, for the Raman imaging of mice injected with PEG-Au-DTTC NPs, the main signal at 506 cm^−1^ was interfered by fluorescence due to the high intensity of noise (Figure 6E,F). This was possibly attributed to the dissociated DTTC molecules in vivo. Using the signal at 847 cm^−1^, the tumor site (arrow 1) can be distinguished with the Raman S/N ratio of 2.71 and confirmed by the histological analysis of the H&E staining (Figure 6G,H).

### 3.7. Therapeutic Efficacy of Intraoperative Raman Image-Guided PTT by PEG-Au-DTTC@CuS NSs

**Figure 7:**
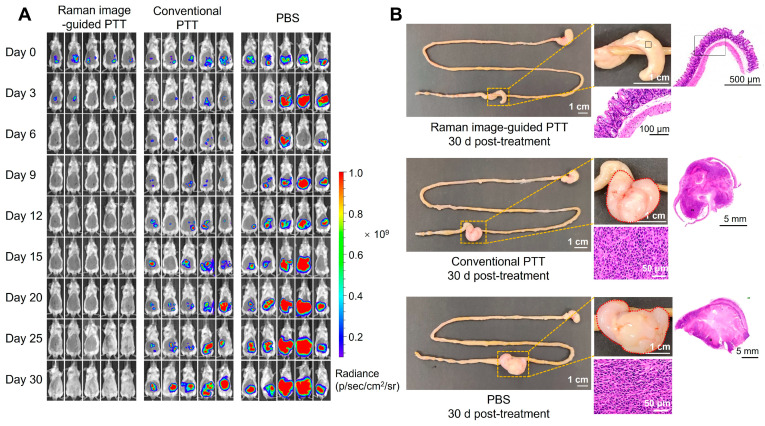
Therapeutic efficacy of the intraoperative Raman image-guided PTT by PEG-Au-DTTC@CuS NSs. (**A**) The in vivo bioluminescence imaging of BALB/c mice bearing orthotopic CT26-Luc colon cancer before (day 0) or after different treatment (*n* = 5). (**B**) Photographs of the resected gastrointestinal tract and histological examination of tumor or cecum at day 30 with H&E staining.

As shown in Figure S9, the Raman image-guided PTT resulted in obvious tumor necrosis throughout the tumor tissue characterized by pyknosis. Mice bearing CT26-Luc orthotopic tumors receiving intraoperative Raman imaging-guided PTT presented a complete cure in 30 days (Figure 7A). The results of H&E staining confirmed that the cecum returned to normal (Figure 7B). By contrast, owing to the incomplete laser irradiation of tumors observed with the naked eye, the mice receiving conventional PTT treatment experienced tumor recurrence after the laser irradiation according to the bioluminescence monitoring.

## 4. Discussion

In this work, PEG was coated on both the Au core and the CuS shell of Au-DTTC@CuS NSs, since we found that the PEG modification was important for maintaining the stability and monodispersion of both nanoparticles. The role of the PEG in the gap between the Au core and CuS shell of the PEG-Au-DTTC@CuS NSs in their biodistribution may be marginal. Besides, the blood distribution of PEG-Au@CuS NSs at 4 h post-injection was significantly lower than that of PEG-AuNPs (Figure S3). Since the particle size of PEG-Au@CuS NSs was double that of PEG-AuNPs (70 nm vs. 35 nm, Figure 1), these results indicated that the particle size may play a more important role in the biodistribution of the PEG-Au@CuS NSs than the pegylation on the surface of the CuS shell.

CuS is considered as a promising coat material. CuS possesses advantages including excellent photoelectric properties and good biocompatibility [41,42]. Besides, coating of CuS layer provides an additional broad absorption in the NIR region peaked at 880 nm, contributing to the enhanced efficacy of cancer PTT with an 808-nm laser. More importantly, CuS NPs facilitated the hepatocyte exocytosis and biliary excretion of Au NPs by copper-transporting ATPase ATP7B. As a result, different structures of the CuS-Au nanoconjugates have been prepared and proven to be promising nanoplatforms for accelerating the elimination of Au nanoparticles [23,30].

Comparatively, silica is commonly used as a coating material owing to its easily modifiable chemistry and good biocompatibility even at a high dose of 100 mg/kg [43]. The mesoporous structure of silica also provides enough conjugation sites for small molecules such as drugs and fluorescent dyes; thus, it is applied to drug delivery, imaging, etc. [44,45,46]. Silica coatings can effectively protect the molecules in the nanoparticles, which is important for in vivo applications [43]. Besides, a silica nanoparticle itself may degrade into nontoxic silicic acid that can be excreted from the body. Thakor et al. reported a gradual decline of Au content in the liver over two weeks post-injection of silica-coated gold nanoparticles [43]. However, there is a paucity of research on how the silica shell affects the metabolism of the gold core.

The excreted Au content in the gallbladders at 4 h in mice injected with PEG-Au@CuS NSs was 19.1-fold that of the PEG-AuNPs of the same particle size of Au NPs. By contrast, in our previous research, for the other two kinds of Au-CuS nanoconjugates, Au content in gallbladders from mice injected with PEG-HCuSNPs@Au was 2.68-fold as high as those injected with PEG-AuNPs; and was 28-fold for PEG-AuNRs@CuS in comparison to those injected with PEG-AuNRs. All the above results illustrated that Au NPs achieved facilitated biliary excretion after the formation of nanoconjugates with CuS NPs. However, the difference in excreted Au content suggested that the clearance ability was associated with the morphology and composition of CuS and Au NPs. Thus, for further improvement of the biliary excretion of Au NPs, attention to the design and optimization of CuS-Au nanoconjugates may help. Besides, other factors such as in vivo stability should also be taken into consideration.

Generally, CuS is not as effective as Au NPs when used as a SERS substrate (Figure S10); thus, they may not produce enough Raman signals for imaging if treated by IV injection. For this reason, we previously injected CuS SERS probes locally into mice tumors prior to surgery for guidance [47]. For CuS NPs, the charge transfer mechanism that formed charge transfer complexes between CuS NPs and DTTC was considered the dominant mechanism for signal enhancement, which is not as efficient as the electromagnetic mechanism for gold-based SERS [48,49]. However, in this work, from Figure 4A it can be seen that for the enhanced stability of Raman reporter molecules, Raman signal intensity was partially sacrificed after the CuS layer coating. Despite the minor decrease in Raman intensity, in vivo Raman imaging revealed that the peak at 506 cm^−1^ and 847 cm^−1^ both performed distinct signals without interference from fluorescence of the dissolved DTTC molecules. Besides, the CuS coating also led to enhanced photothermal effects in this work. All in all, an appropriate layer structure should be considered in the design of the Raman probe to achieve better in vivo efficacy.

## 5. Conclusions

In this work, we prepared PEG-AuNPs and core–shell-structured PEG-Au@CuS NSs. Thanks to the CuS coating, PEG-Au@CuS NSs exhibited significantly increased hepatobiliary excretion in comparison to PEG-AuNPs. The percent of Au exocytosis from the primarily cultured mouse hepatocytes following the 5 min uptake of PEG-AuNPs and PEG-Au@CuS NSs was 46% and 88%, respectively. Au content in gallbladders from mice injected with PEG-Au@CuS NSs was 8.2-fold and 19.1-fold as high as those injected with PEG-AuNPs at 1 h and 4 h, respectively. On the other hand, PEG-Au@CuS NSs performed an increased UV absorption at NIR wavelengths as well as improved photothermal efficacy compared with PEG-AuNPs. After the DTTC loading, the synthetic PEG-Au-DTTC@CuS NSs achieved intraoperative tumor Raman imaging with a high S/N ratio. The combination of the precise detection of the tumor site and the intense photothermal conversion effect of PEG-Au-DTTC@CuS NSs offered a complete cure for mice bearing colon cancer. Our results demonstrated that PEG-Au-DTTC@CuS NSs held great promise for intraoperative Raman image-guided tumor PTT, especially for colon cancer phototheranostics. This work provides an approach to designing an Au-based PTT agent that is capable of SERS image guidance and accelerated in vivo clearance.

## Data Availability

The data presented in this study are available on request from the corresponding author.

## Supplementary Materials

The following supporting information can be downloaded at: https://www.mdpi.com/article/10.3390/pharmaceutics16081089/s1, Figure S1: The size distribution of PEG-Au@CuS NSs following incubation for different times in 10% FBS at 37 °C. Figure S2: Cellular Au and percent of Au exocytosis from the primarily cultured Kupffer cells following the 5 min uptake of PEG-AuNPs or PEG-Au@CuS NSs. Figure S3: Biodistribution profile of Au in blood and major organs of mice at 4 h after IV injection of PEG-Au@CuS NSs or PEG-AuNPs. Figure S4: Photothermal effect of PEG-AuNPs or water at different concentrations under 808 nm laser irradiation for 10 min. Figure S5: Temperature–time curves of PEG-Au@CuS NSs or PEG-AuNPs and time constant of heat transfer. Figure S6: Zeta potential of PEG-Au-DTTC NPs and PEG-Au-DTTC@CuS NSs. Figure S7: Cell viability of NIH 3T3 cells after incubation with PEG-Au-DTTC@CuS NSs at various concentrations of Au for 24 h. Figure S8: Cell viability of CT26-Luc cells treated with PEG-Au-DTTC@CuS NSs for 4 h followed by irradiation using 808-nm laser (1.0 W/cm^2^, 5 min) or without laser (dark) and incubated for another 12 h. Figure S9: Micrographs of H&E staining of tumor section of mice collected at 24 h after Raman image-guided PTT. Figure S10: Raman spectra of PEG-Au-DTTC NPs, PEG-Au-DTTC@CuS NSs and PEG-CuS-DTTC NPs at a concentration of 0.35 μg/mL of DTTC, respectively. Reference [50] is cited in the Supplementary Materials.
