# Supplementary material for: Au@CuS Nanoshells for Surface-Enhanced Raman Scattering Image-Guided Tumor Photothermal Therapy with Accelerated Hepatobiliary Excretion
Source: Pharmaceutics. 2024 Aug 20;16(8):1089. doi: 10.3390/pharmaceutics16081089 (PMC11360001; doi:10.3390/pharmaceutics16081089)
Supplement: Supplementary file 1 [file pharmaceutics-16-01089-s001.zip › pharmaceutics-3082816-supplementary.pdf]

Supplementary Information

Au@CuS Nanoshells for Surface-Enhanced Raman Scattering Image-Guided Tumor Photothermal Therapy with Accelerated Hepatobiliary Excretion

Zhang S. et al.

Supplementary methods

Size distribution in FBS

PEG-Au@CuS NSs were added to 10% FBS under 37 °C at a concentration of 0.1 mg/mL. DLS analysis was performed at 0, 6, 12, and 24 h following incubation.

Calculation of the photothermal conversion efficiency

The photothermal conversion efficiency (η) is calculated as follows [50]:

$$\eta = \frac{hS(T_{Max} - T_S) - Q_C}{I(1 - 10^{-A_{808}})} \quad \text{(S1)}$$

where h represents heat transfer coefficient; S represents the surface area of the container; Q_C represents heat dissipated from light absorbed by the quartz sample cell itself; T_{Max} represents the maximum system temperature; and T_S represents ambient temperature. The ($T_{Max}-T_S$) of PEG-Au@CuS NSs and PEG-AuNPs aqueous solution is 27.2°C and 7.8°C according to Figure S5A, respectively. Laser power intensity (I) is 1.0 W/cm^2. The absorbance of PEG-Au@CuS NSs and PEG-Au NPs aqueous solution at 808 nm (A_{808}) is 0.4 and 0.0425, respectively.

In vivo biodistribution analysis

BALB/c mice were IV-injected with PEG-AuNPs or PEG-Au@CuS NSs (40 mg/kg of Au) and euthanized at 4 h (n = 3). Blood and major organs were collected and weighed. After being digested with aqua regia, the Au content was quantified by ICP-OES.

Supplementary figures

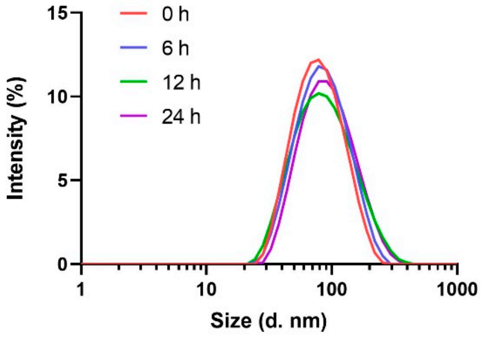

Figure S1. The size distribution of PEG-Au@CuS NSs following incubation for different times in 10% FBS at 37 °C.

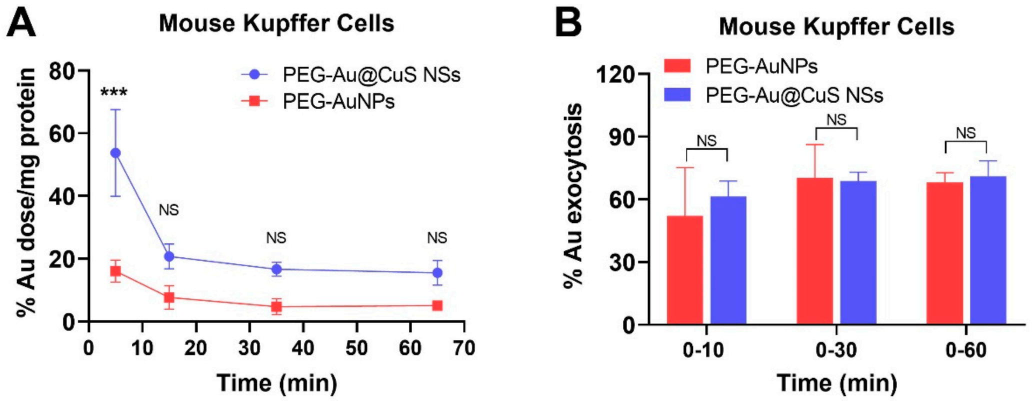

Figure S2. Cellular Au (**A**) and percent of Au exocytosis (**B**) from the primarily cultured Kupffer cells following the 5 min uptake of PEG-AuNPs or PEG-Au@CuS NSs, respectively. Data are presented as mean ± SD (n = 3). Statistical significance was calculated by two-way ANOVA with Sidak's post-hoc test. *** $p < 0.001$, NS, no significant difference.

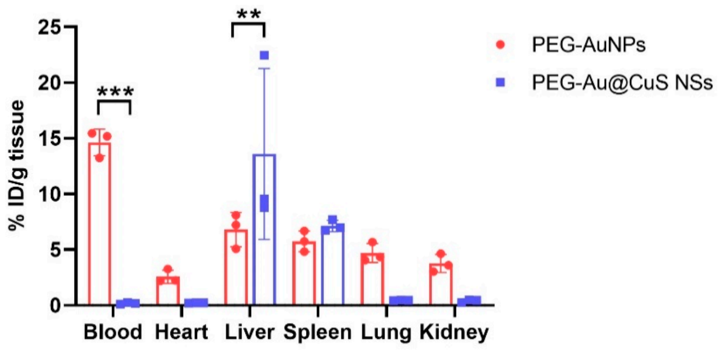

Figure S3. Biodistribution profile of Au in blood and major organs of mice at 4 h after IV injection of PEG-Au@CuS NSs or PEG-AuNPs (40 mg/kg of Au). % ID/g tissue, percentage of the injected dose per gram tissue. Data are presented as mean ± SD (n = 3). Statistical significance was calculated by two-way ANOVA with Sidak's post-hoc test. ** $p < 0.01$, *** $p < 0.001$.

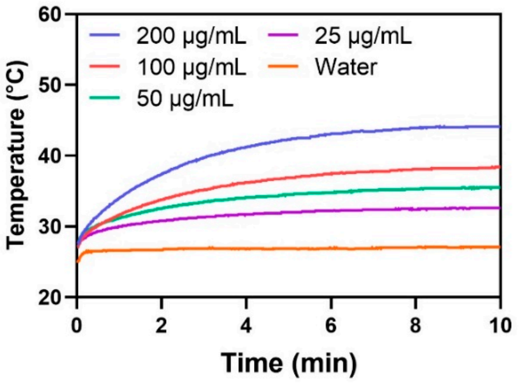

Figure S4. Photothermal effect of the PEG-AuNPs solution at different concentrations of Au or water under 808-nm laser irradiation (1.0 W/cm^2) for 10 min.

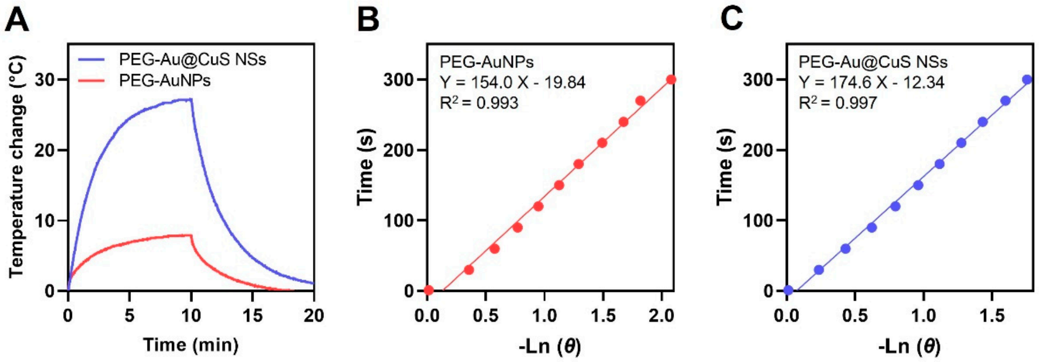

Figure S5. (**A**) Temperature–time curves of PEG-Au@CuS NSs or PEG-AuNPs (50 µg/mL of Au). (**B**,**C**) Time constant of heat transfer from PEG-AuNPs or PEG-Au@CuS NSs was determined to be 154 s or 175 s by applying the linear time data from the cooling period versus the negative natural logarithm of driving force temperature, which was obtained from (**A**).

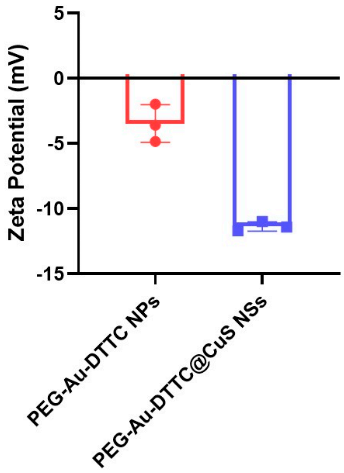

Figure S6. Zeta potential of PEG-Au-DTTC NPs and PEG-Au-DTTC@CuS NSs. Data are presented as mean ± SD (n = 3).

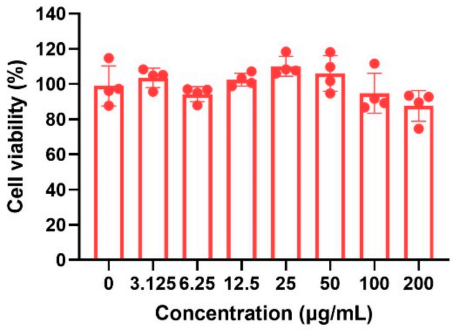

Figure S7. Cell viability of NIH 3T3 cells after incubation with PEG-Au-DTTC@CuS NSs at various concentrations of Au for 24 h. Data are presented as mean ± SD (n = 4).

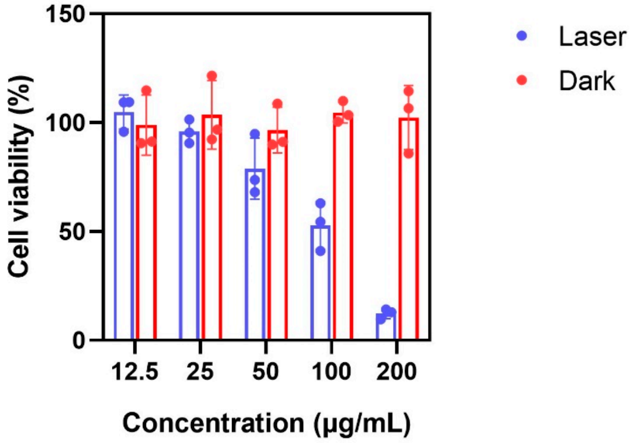

Figure S8. Cell viability of CT26-Luc cells treated with PEG-Au-DTTC@CuS NSs for 4 h followed by irradiation with an 808-nm laser (1.0 W/cm^2, 5 min) or without laser (dark). The cells were incubated for another 12 h. Data are presented as mean ± SD (n = 3).

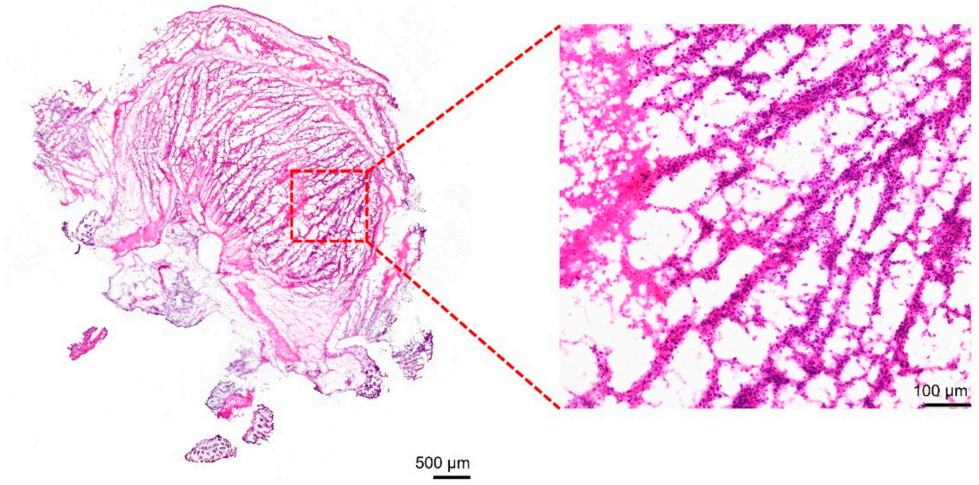

Figure S9. Micrographs of H&E staining of tumor section of mice collected at 24 h after Raman image-guided PTT in Figure 7A.

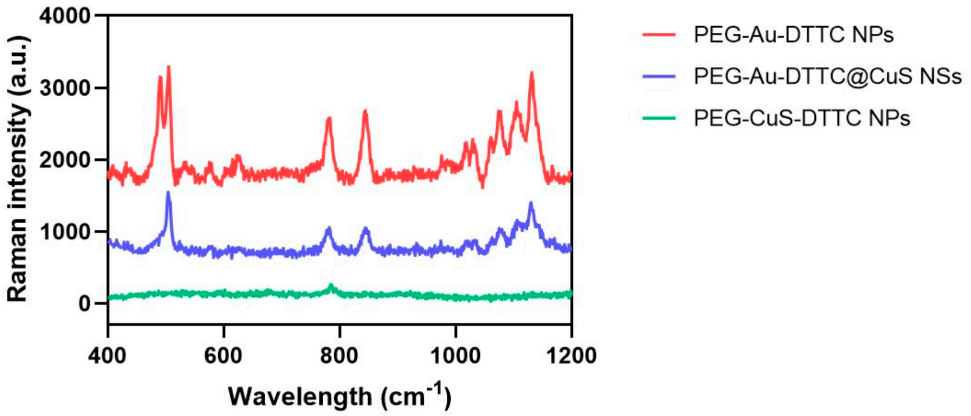

Figure S10. Raman spectra of PEG-Au-DTTC NPs, PEG-Au-DTTC@CuS NSs and PEG-CuS-DTTC NPs at a concentration of 0.35 μg/mL of DTTC, respectively.

Reference

50. Roper, D.K.; Ahn, W.; Hoepfner, M. Microscale heat transfer transduced by surface plasmon resonant gold nanoparticles. *J. Phys. Chem. C* **2007**, *111*, 3636-3641.
